# Effect of Early Ciprofloxacin Administration on Growth Performance, Meat Quality, Food Safety, and Metabolomic Profiles in Xueshan Chickens

**DOI:** 10.3390/ani14162395

**Published:** 2024-08-18

**Authors:** Lan Huang, Jialuo Sun, Qixin Guo, Yong Jiang, Bai Hao, Guobin Chang

**Affiliations:** 1 Key Laboratory for Animal Genetics & Molecular Breeding of Jiangsu Province, College of Animal Science and Technology, Yangzhou University, Yangzhou 225009, China; dx120200133@stu.edu.cn (L.H.); 531603755@163.com (J.S.); dx120190114@yzu.edu.cn (Q.G.); jiangyong12126@163.com (Y.J.); 2 Joint International Research Laboratory of Agriculture and Agri-Product Safety, The Ministry of Education of China, Institutes of Agricultural Science and Technology Development, Yangzhou University, Yangzhou 225009, China

**Keywords:** ciprofloxacin, chicken, meat quality, metabolomics, WGCNA

## Abstract

**Simple Summary:**

The widespread use of antibiotics in poultry production has raised public concerns about food safety and meat quality. This study investigated the effects of early ciprofloxacin use on Xueshan chickens, focusing on growth, meat quality, and metabolic changes. It was found that, while ciprofloxacin treatment did not affect overall chicken growth, it significantly altered meat quality characteristics, which may affect meat texture and preservability. Although ciprofloxacin residues were not detected in muscle, metabolic analyses revealed significant metabolic alterations, particularly in microbial and amino acid metabolism pathways. These findings emphasize that antibiotic use may have far-reaching effects on poultry product quality, involving not only direct meat quality changes but also complex metabolic reactions. This study provides new perspectives for understanding the role of antibiotics in poultry production, which is important for optimizing antibiotic-use strategies and ensuring food safety. The results may be an important reference for poultry producers, food safety regulators and consumers.

**Abstract:**

To investigate the effects of early administration of ciprofloxacin (CIP) on Xueshan chickens, in this study Xueshan chickens were measured for growth performance, tested for drug residues, evaluated for meat quality, and muscle metabolism changes were explored using a non-target metabolomics approach. Experimental findings revealed that early CIP use did not significantly impact the overall growth rate of Xueshan chickens (*p* > 0.05). However, notable alterations in meat quality were observed: the CIP-treated group exhibited a significant decrease in muscle pH (pH_1_ and pH_24_) and a marked increase in drip loss and moisture content (*p* > 0.05). No CIP residues were detected in muscle tissue. Untargeted metabolomics analyses unveiled significant alterations in the metabolic profile of market-age chickens following CIP treatment. Both functional enrichment and metabolic network analyses indicated significant effects on the ko01120 (microbial metabolism in diverse environments) and ko00350 (tyrosine metabolism) pathways, implying that CIP treatment may influence chicken meat quality by modulating microbial communities and amino acid metabolism. This study provides a crucial foundation for understanding the impact of antibiotics on meat quality and metabolism in poultry production, offering scientific insights for optimizing antibiotic-use strategies and safeguarding poultry product quality.

## 1. Introduction

Antimicrobial agents play a pivotal role in contemporary animal husbandry due to their capacity to inhibit or eliminate micro-organisms [1]. Intensive livestock production has emerged as the predominant consumer of antibiotics globally, accounting for 73% of total usage [2]. These antimicrobials are primarily employed to promote animal growth and to prevent and treat diseases [3]. However, the widespread utilization of antibiotics has raised numerous concerns, including issues related to antibiotic residues [4,5], antimicrobial resistance [6,7], and potential effects on animal physiology and metabolism [8]. Consequently, a comprehensive assessment of the impact of antibiotic use in livestock and poultry farming is crucial for developing scientifically sound regulations on antibiotic usage and safeguarding public health and safety [9].

Current research predominantly focuses on the effects of long-term antibiotic use in animals [8]. For instance, studies have demonstrated that antibiotics can enhance animal performance by influencing core cecal micro-organisms and disrupting host microbiota metabolism [8]. Additionally, research has indicated that prolonged antibiotic use may exacerbate the transmission of resistance genes from farms to human populations [10,11,12]. In contrast, relatively few studies have investigated the early prophylactic use of antibiotics. One study by Jankowski et al. suggests that early administration of antibiotics may suppress humoral immunity in chickens [13]. Nevertheless, there remains a dearth of research examining the effects of early antibiotic use on the quality of livestock and poultry products.

Ciprofloxacin (CIP), a broad-spectrum fluoroquinolone antibiotic, was widely used in livestock and poultry farming [14,15] Considering that poultry is an important source of animal protein and its industry has grown faster than any other source of animal protein over the past half century [16], it is particularly important to study the effects of antibiotics on poultry. Xueshan chickens [17], a speciality poultry breed, is popular among consumers for its excellent meat quality. Therefore, it is not only scientifically important to explore the effects of early CIP use on the quality and metabolic profiles of c, but there are also implications for food safety and quality.

As an emerging system biology research method, untargeted metabolomics can comprehensively reflect the changes in the metabolic state of the organism, providing a powerful tool for in-depth understanding of the effects of antibiotics on animal physiology and metabolism [18,19]. Compared with traditional targeted analysis methods, untargeted metabolomics can detect hundreds or even thousands of metabolites simultaneously, thus providing a more comprehensive metabolic profile [20]. This approach is not only capable of discovering known changes in metabolic pathways, but may also reveal new metabolic markers and unknown biological mechanisms. Weighted gene co-expression network analysis (WGCNA) is an advanced bioinformatics method originally developed for genomics but now widely applied in metabolomics [21,22]. This technique constructs networks of metabolites based on their co-expression patterns across different samples or conditions [23]. This approach provides a more holistic view of metabolic alterations induced by antibiotics, potentially revealing unexpected effects and mechanisms of action that might be missed by traditional analytical methods.

Based on the above background, the aim of this study is to investigate the comprehensive effects of early ciprofloxacin use on Xueshan chickens, including growth performance, conventional meat quality, drug residues, and chicken metabolic profiles. Focusing on metabolic changes, we will delve into the effects of ciprofloxacin treatment on the metabolic profiles of Xueshan chickens through an untargeted metabolomics approach combined with multivariate statistical analysis and weighted gene co-expression network analysis (WGCNA).

## 2. Materials and Methods

### 2.1. Ethics Statement

The animal experiments conducted in this study were reviewed and approved by the Animal Care and Use Committee of Yangzhou University (Approval No. SYDW-2019015).

### 2.2. Animals and Sampling

A total of 180 healthy male Xueshan chickens from Yangzhou Lihua Livestock and Poultry Co., Ltd. (Yangzhou China) were selected for the experiment. Xueshan chickens represent an important meat-type yellow-feathered breed in China which have been meticulously developed through the crossbreeding of high-quality local Tibetan chickens and Chahua chickens [24]. The chicks were randomly allocated to two groups (three pens per group) at one day of age. Between 14 and 16 days of age, the experimental group was administered water containing CIP (0.6 g/L, dosage based on the drug instruction manual), while the control group was provided with plain water. The chickens were reared on the floor until they reached the age of marketability (98 days), with unrestricted access to feed and water throughout the study period.

At the age of market maturity, samples of thigh muscle (30 samples in each group) were obtained from slaughtered chickens. The samples were divided into two portions: thigh muscles other than the quadriceps were stored at 4 °C for meat quality analysis, while the quadriceps were immediately frozen and stored at −80 °C for subsequent mass spectrometry analysis.

### 2.3. Growth Performance and Meat Quality

At 19, 23, 28, 33, 43, 58, 78, and 98 days of age, 30 males were randomly selected from each group, and weighed with a portable electronic scale (Kaifeng, Jinghua, China). These measurements were utilized to plot growth curves for groups.

Color, pH, rate of drip loss, and shear force of the meat were all assessed in the left thigh muscles (30 samples in each group). The pH was measured at 1 h and 24 h (pH_1_ and pH_24_, after storing the muscle at 4 °C) post-mortem using a pH meter (pH-STAR, Matthaus, Berlin, Germany) calibrated with pH 4.01 and 7.01 buffers. Three readings were taken, and the average pH was calculated. The drip loss rate was measured using a meat-quality pressure meter (Meat-1, Tenovo Food, Beijing, China) according to the method described by Li et al. [25]. Meat samples (0.125 cm^3^) were wrapped in absorbent paper and placed into the machine for testing. The program was set to 300 N for 5 min, and each sample was measured three times, with the final result calculated as the average. To determine drip loss, muscle samples were cut into strips (1.0 cm width × 0.5 cm thickness × 2.5 cm length), weighed (WTM), stored in plastic bags, and cooked in a water bath at 80 °C for 15 min. After cooling to room temperature, the samples were gently patted dry with paper towels and weighed again (WTC). The cooking loss was calculated as [(WTM − WTC)/WTM] × 100.

Muscle lightness (L*), redness (a*), and yellowness (b*) were determined using a chroma meter (CR-400, Konica Minolta, Tokyo, Japan) according to the CIELAB color system, where L* ranges from 0 (black) to 100 (white), a* from green to red, and b* from blue to yellow [17]. The sample surfaces were freshly trimmed and free of fat or connective tissue.

The proximate composition of the left breast and muscle samples was determined using a near-infrared spectrophotometer with an artificial neural network calibration model and database (FOSS FoodScan 78800, Dedicated Analytical Solutions, Hilleroed, Denmark). All exterior fat and connective tissue were removed prior to analysis. Each sample was coarsely ground using a tabletop grinder to obtain a sample of approximately 180 g, which was then placed in a 140 mm round sample dish and analyzed in the FoodScan. The final reported values were calculated by averaging three independent readings for each sample and included the percentages (g/100 g) of protein, intramuscular fat, collagen, and moisture [25].

### 2.4. Detection of Drug Residues

The CIP content in thigh muscles (30 samples in each group) was determined by the LC-MS/MS (Liquid Chromatography-Massspectrometry/Mass Spectrometry) method with reference to the National Standard GB 31658.17-2021 [26] of the People’s Republic of China.

### 2.5. Untargeted Metabolomics

Twelve bicep femoris samples were used for untargeted metabolomic assays (6 samples per group). Samples were slowly thawed at 4 °C, then a portion was added to a pre-cooled methanol/acetonitrile/water solution (2:2:1, *v*/*v*), vortexed, and sonicated at low temperature for 30 min. After standing at −20 °C for 10 min, the samples were centrifuged at 14,000× *g* and 4 °C for 20 min. The supernatant was collected, dried under vacuum, and re-dissolved in an acetonitrile/water solution (1:1, *v*/*v*) for mass spectrometry analysis. The samples were vortexed and centrifuged again at 14,000× *g* and 4 °C for 15 min before analysis.

Liquid chromatography-mass spectrometry (LC-MS) analysis was performed using an Agilent 1290 Infinity UHPLC system. Samples (2 μL injection volume) were separated on an Agilent HILIC column at 25 °C with a flow rate of 0.5 mL/min. Primary and secondary mass spectra were collected using an AB Triple TOF 6600 mass spectrometer.

### 2.6. Analysis of Metabolomic Data

Positive ion mode (POS) and negative ion mode (NEG) are both used to detect metabolites, which can make the metabolite coverage higher and the detection effect better. In the process of subsequent data analysis, the positive and negative ion models are analyzed mixed.

PLS-DA (partial least squares discriminant analysis) and OPLS-DA (orthogonal projection to latent structures-discriminant analysis) were applied in comparison groups using R package ropls (http://www.rproject.org/, accessed on 17 June 2024) [27]. A variable importance in projection (VIP) score of OPLS model was applied to rank the metabolites that best distinguished between two groups. The threshold of VIP was set to 1. The abundances of differential metabolites were normalized by z-score and hierarchically clustered by R package pheatmap (https://github.com/raivokolde/pheatmap, accessed on 17 June 2024) to show the accumulation differences between two groups. KEGG (Kyoto Encyclopedia of Genes and Genomes) is the major public pathway-related database that includes not only genes but metabolites [28]. Metabolites were mapped to KEGG metabolic pathways for annotation and enrichment analysis. The network plot for hub pathway was conducted by cytoscape [29].

### 2.7. Weighted Gene Co-Expression Network Analysis

To improve the accuracy of network construction, the selected gene set was first screened and filtered to remove low-quality metabolites or samples that affect the results. The WGCNA R package was used to construct a weighted gene co-expression network [22,30]. An adjacency matrix was constructed according to the scale-free network.

### 2.8. Important Metabolite Screening and Functional Annotation

Pearson’s correlation coefficient was calculated between each module eigengene (ME) and the treatment group (CIP-treated vs. control), and tested for significance [22]. Based on the *p*-values and correlation coefficients, modules significantly associated with the CIP treatment were identified. Within these significant modules, potential key metabolites were screened by calculating the module membership (MM) of each metabolite. Metabolites with MM > 0.8 were considered highly connected to the module and potentially important in the response to CIP treatment. Metabolic pathway annotation and functional enrichment analysis of identified key metabolites were conducted using KEGG. The network plot for the hub pathway was done by cytoscape [29].

### 2.9. Statistical Analysis

Statistical analysis was performed using the SPSS 25.0 software (SPSS Inc., Chicago, IL, USA) and the analysis of two-group comparisons using Student’s *t*-test. *p* < 0.05 (*) and *p* < 0.01 (**) represent statistical significance, and all data are shown as means ± SD (standard deviation).

## 3. Results

### 3.1. Growth Curves and Meat Quality

**Figure 1:**
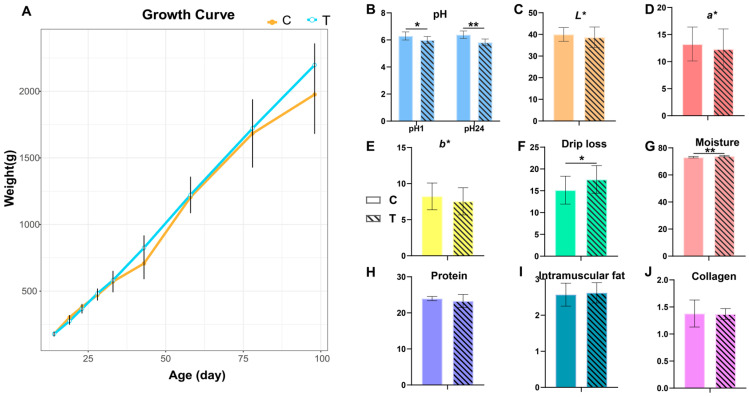
Growth performance and meat quality of control and CIP-treated chickens. (**A**) Growth curve: this graph illustrates the body weight progression of chickens over time, comparing the control group (C) with the CIP-treated group (T). (**B**–**J**) pH_1_, pH_24_, L*, a*, b*, drip loss, moisture, protein, intramuscular fat, and collagen. C: control group; T: CIP-treated group. Subsequent figures are also represented in this way. *: *p* < 0.05, **: *p* < 0.01.

The analysis involved the statistical examination of the data on body weight and traditional meat quality (Tables S1 and S2, Figure 1). The findings revealed that there were no notable discrepancies in body weight (Figure 1A) between the two cohorts until they reached the market-age stage (*p* > 0.05). However, distinct variations were observed in the muscle metrics including pH_1_, pH_24_, drip loss, and moisture. In the group that received medication, both pH_1_ and pH_24_ experienced a notable decrease (*p* < 0.05), whereas drip loss and moisture showed significant increments (*p* < 0.05).

### 3.2. Detection of Ciprofloxacin

Liquid chromatography-tandem mass spectrometry (LC-MS/MS), a highly sensitive and specific analytical technique, was used to quantify CIP residues in chicken tissue. The results of the analysis showed that the concentration of CIP in muscle tissue was below the limit of detection of the method, as shown in Table S3. This result suggests that CIP is effectively metabolized or eliminated in muscle tissue, and that early use of this antimicrobial does not pose a significant drug residue risk to marketed chicken meat. However, it is important to note that, although no CIP residues were detected, further studies are necessary to investigate the possible presence of metabolites or transformation products in the tissues.

### 3.3. Early Feeding with Ciprofloxacin Altered the Metabolic Profile of Chicken Meat

**Figure 2:**
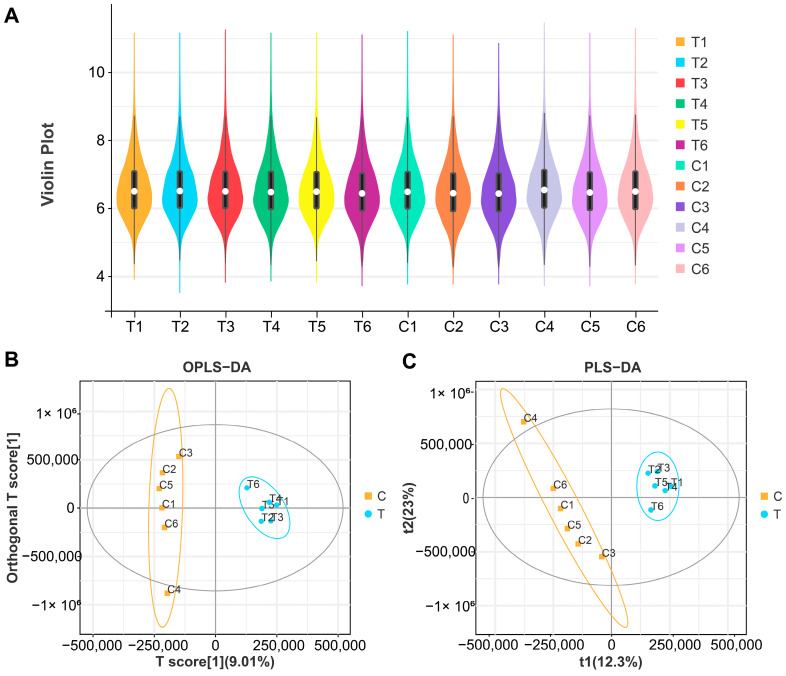
CIP alters the metabolic profile of chicken. (**A**) Violin plot of samples: this violin plot illustrates the distribution of metabolite intensities or concentrations across all samples from both the control and CIP−treated groups. (**B**) Analysis of OPLS−DA (orthogonal partial least squares discriminant analysis). (**C**) Analysis of PLS−DA (partial least squares discriminant analysis).

To examine the impact of CIP on chicken meat metabolism, this research conducted untargeted metabolomic analysis on market-age chicken meat from experimental and control groups. The analysis involving the overlay of TIC (total ion chromatograms) from mass spectrometry detection of QC (quality control) samples is shown in Figure S1. The research results demonstrate that the total ion flows of metabolites exhibit a high degree of overlap, with chromatographic peaks showing approximately the same retention times and response intensities. This indicates that experimental detections performed on the same sample at different times possess good stability, thus lending credibility to the data results. A total of 19,080 metabolites were identified in 12 samples, including 1229 known metabolites detected through POS (positive ion mode) and 1058 through NEG (negative ion mode) (Table S4). Figure 2A illustrates the composite metabolite data profile per sample (Figure 2A). Both OPLS-DA scores and PLS-DA results for samples and metabolites revealed significant discrepancies between the test and control groups (Figure 2B,C).

**Figure 3:**
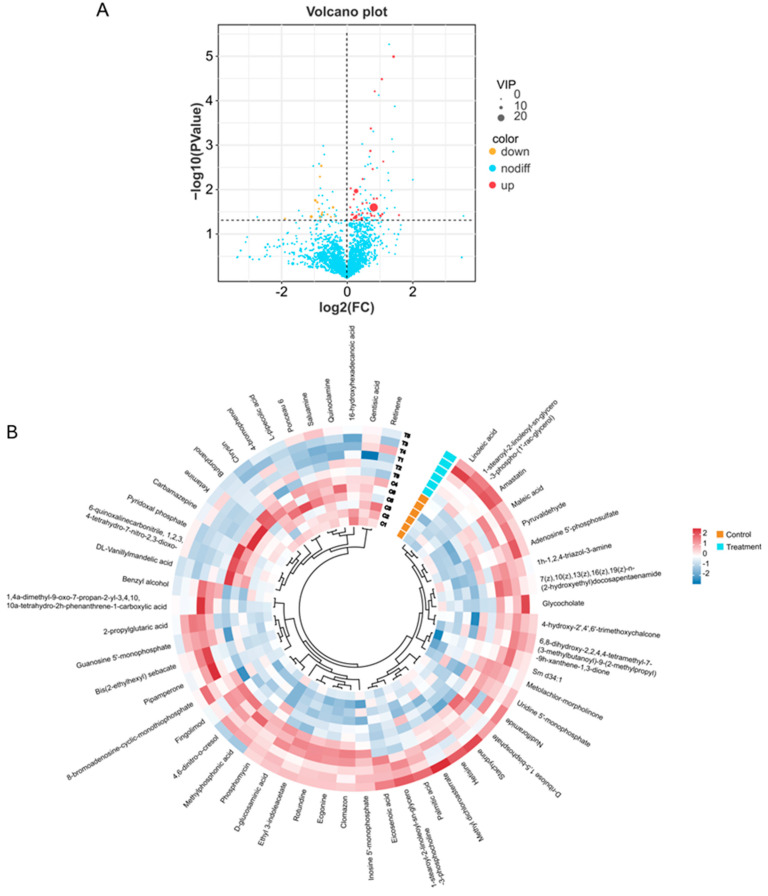
Analysis of differential metabolites between control and CIP−treated chicken breast meat. (**A**) Volcano plot of differential metabolite. (**B**) Circular heat map of differential metabolite.

Moreover, a blend of *t*-test analysis and variable importance in projection (VIP) values (*p* < 0.05, VIP > 1) was utilized to pinpoint distinct metabolites between the two groups. The results indicated that after CIP treatment, 39 metabolites showed an increase, while 15 metabolites exhibited a decrease (Figure 3A). The circular heatmap visualization elucidates the distribution of metabolites, effectively illustrating the previously delineated distinctions among the samples (Figure 3B). Upon examination of the graphical representation, a clear dichotomization of metabolite clusters is evident, corresponding to the two predefined categories. This spatial arrangement of metabolites within the circular heatmap provides a robust visual confirmation of the metabolomic disparities between the sample groups, facilitating a more nuanced interpretation of the underlying biochemical variations.

**Figure 4:**
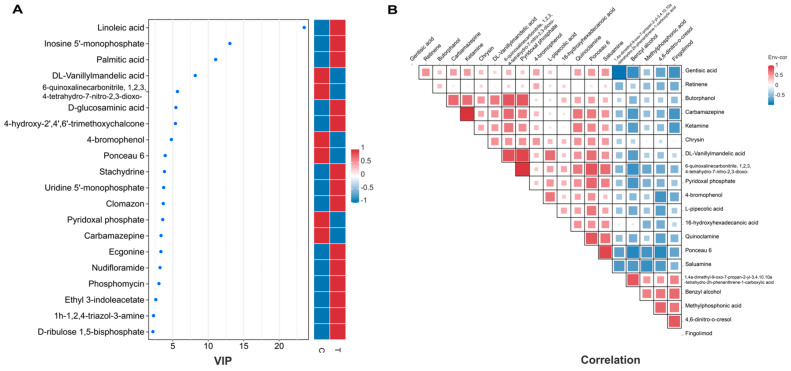
Comparative analysis of metabolite profiles and their correlations under control and CIP treatment conditions in chicken. (**A**) Comparative analysis of metabolite profiles. (**B**) Correlations of metabolites under control and CIP conditions.

Figure 4A illustrates the top 20 differential metabolites for VIP values. The magnitude of the VIP score is directly proportional to the metabolite’s contribution to sample differentiation. Metabolites exhibiting elevated VIP values are frequently prioritized as potential biomarker candidates, meriting further investigation and validation. It was found that Linoleic acid, Inosine 5′-monophosphate, Palmitic acid, D-glucosaminic acid, and 4-hydroxy-2′, 4′, 6′-trimethoxychalcone were potential biomarkers of the test group. DL- Vanillylmandelic acid, 6-quinoxalinecarbonitrile, 1,2,3,4-tetrahydro-7-nitro-2,3-dioxo, 4-romophenol, Pyridoxal phosphate, and Ponceau 6, among others, were potential biomarkers of the control group. Correlation analysis of differential metabolites was also performed in this study and the results are shown in Figure 4B. DL-Vanillylmandelic acid significantly correlates with 6-quinoxalinecarbonitrile, 1,2,3,4-tetrahydro-7-nitro-2,3-dioxo, Pyridoxal phosphate, and Ponceau 6.

These multivariate statistical analyses provide robust evidence for distinct metabolic profiles between CIP−treated and untreated chicken meat samples, suggesting a substantial impact of CIP treatment on the metabolome of market-aged chicken meat.

### 3.4. Functional Analysis of Differential Metabolites

**Figure 5:**
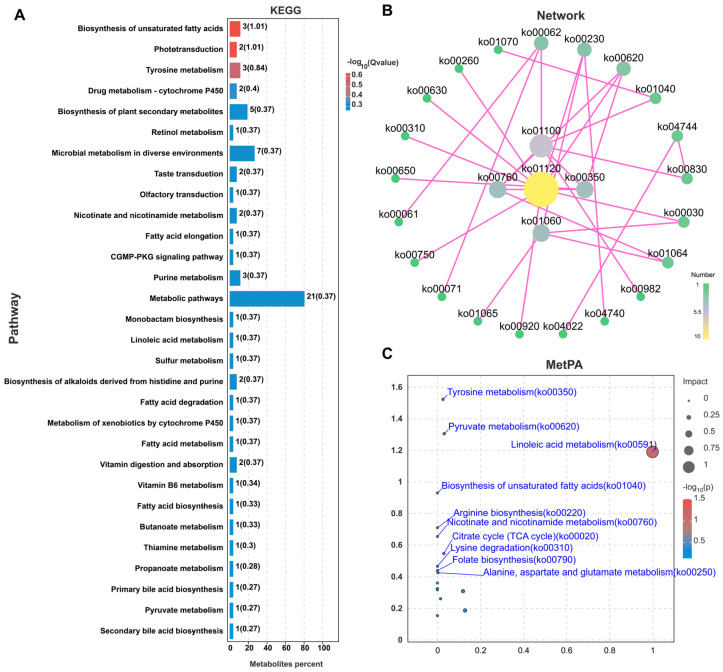
Functional analysis of differential metabolites in chicken breast meat under CIP treatment. (**A**) Top 30 of KEGG enrichment. (**B**) Network plot of enriched pathways. (**C**) Metabolomic Pathway Analysis.

KEGG functional enrichment analysis was performed on metabolites that exhibited significant differences between the two groups. The results were visualized using a bar plot (Figure 5A). Our findings indicate that early administration of CIP primarily affected several metabolic pathways in Xueshan chicken hen meat, including Arachidonic acid metabolism, Fc epsilon RI signaling pathway, Purine metabolism, Oxytocin signaling pathway, Pyrimidine metabolism, Serotonergic synapse, and various metabolic pathways.

Metabolic pathway networks mapped with Cytoscape depicted the intricate interactions among metabolites (Figure 5B). The network analysis revealed several highly interconnected functional modules, including ko01120 (Microbial metabolism in diverse environments), ko00350 (Tyrosine metabolism), ko01100 (metabolic pathways), ko00760 (Nicotinate and nicotinamide metabolism), and ko01060 (biosynthesis of plant secondary metabolites). These modules are primarily involved in microbial metabolism, amino acid metabolism, energy metabolism, and secondary metabolism, among others. These findings provide valuable insights into understanding systemic changes in metabolic networks and help elucidate complex metabolic regulatory mechanisms.

Using the nomenclature and abundance information of the differential metabolites in the comparison group, along with the KEGG database as a background, MetPA (metabolomics pathway analysis) was conducted. The principle of “betweenness centrality” was applied to calculate the positional information of the pathways in the regulation of the metabolite network, targeting the important metabolic pathways involving the differential metabolites in the comparison group. The results revealed that the pathways enriched by MetPA were primarily Tyrosine metabolism, Linoleic acid metabolism, Pyruvate metabolism, Biosynthesis of unsaturated fatty acids, Citrate cycle (TCA cycle), and Nicotinate and nicotinamide metabolism.

### 3.5. Overview of Metabolite-Weighted Co-Expression Network Analysis (WGCNA) Results

**Figure 6:**
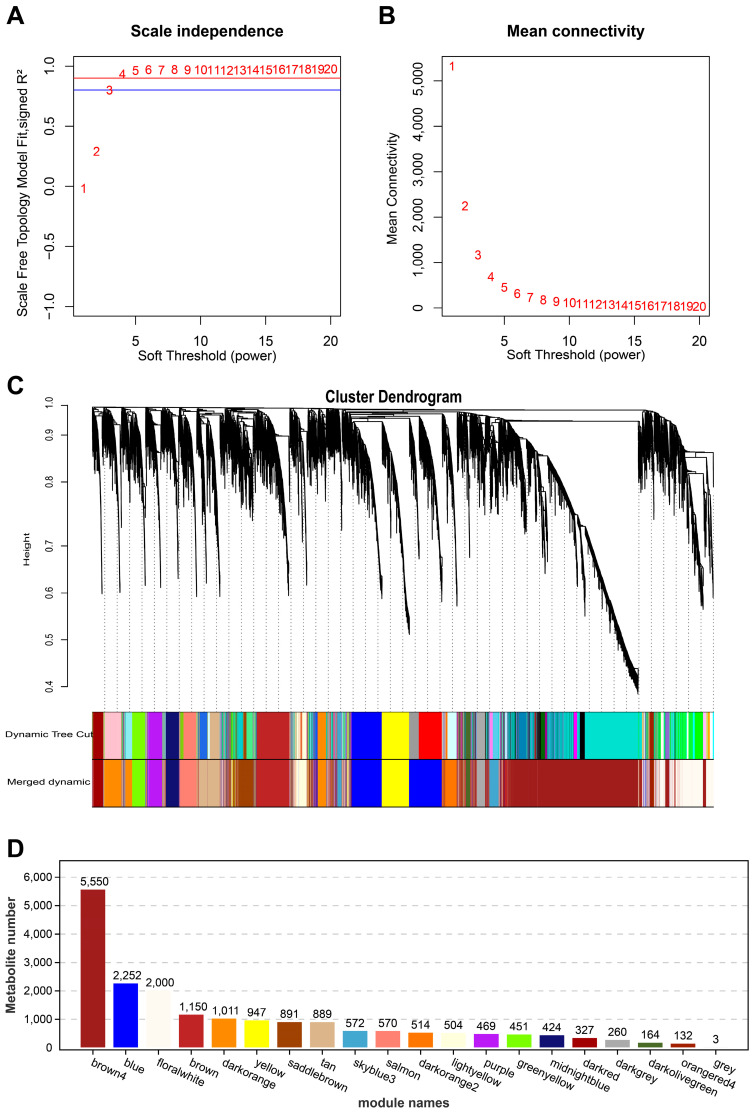
WGCNA analysis of metabolite profiles in chicken under control and CIP treatment conditions. (**A**) Sample detection and the selection and validation of the optimal soft threshold power for constructing gene co-expression networks. (**B**) Scale independence and mean connectivity for the WGCNA analysis of all samples. (**C**) Clustering dendrograms of all samples. (**D**) The number of metabolites in each module.

WGCNA revealed modular structure and scale-independent features of metabolites. The soft threshold was selected based on the Scale Free Topology Model Fit and Mean Connectivity. As shown in Figure 6A, the scale-independent topology fit index R^2^ reached above 0.9 when the soft threshold power value was 4, while maintaining a high Mean Connectivity (Figure 6B). The clustering dendrogram and dynamic shear algorithm identified 23 distinct metabolite modules (Figure 6C). The size of these modules varied significantly, ranging from 3 to 5550 metabolites. Among them, the brown module (brown) was the largest, containing 5550 metabolites, while the blue module (blue) was the second largest, containing 2252 metabolites. There are also several medium-sized modules such as brown4 with 2000 metabolites, floral-white with 1150 metabolites, and dark orange with 1001 metabolites (Figure 6D). This modular structure reflects the complexity and hierarchical nature of the metabolic network and provides a basis for further exploration of functional associations between metabolites.

### 3.6. Construction of Module–Trait Relationships and Detection of Key Modules

**Figure 7:**
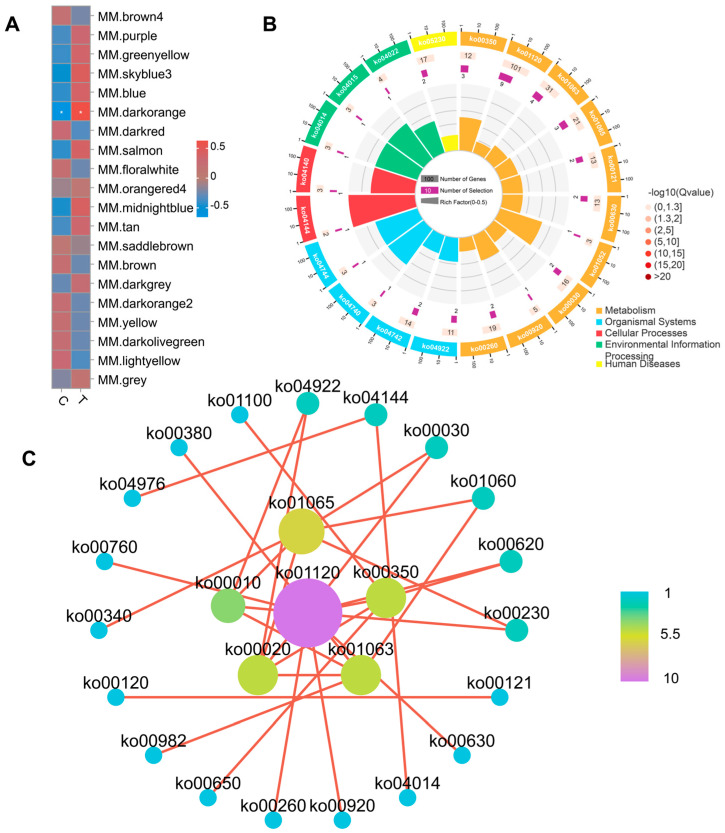
Key module screening and functional analysis of metabolite co-expression networks in chicken under CIP treatment. (**A**) The relationship of module and trait. Asterisks (*) indicate statistically significant differences (*p* < 0.05). (**B**) KEGG enrichment analysis for the metabolites in dark orange module. (**C**) Network plot of enriched pathways.

In order to find the key metabolic modules, the present study analyzed the correlation of the modules with the traits using Pearson’s correlation coefficient (Figure 7A). The results showed that dark orange modules showed significant correlation with CIP treatment. The differential metabolites in the dark orange module were analyzed by KEGG enrichment, and the metabolic pathways with *p* value Top25 are shown in the circle diagram (Figure 7B).

The metabolic pathway network diagram constructed using Cytoscape visualized the complex interactions between different metabolites (Figure 7C). Network analysis again revealed several highly interconnected functional pathways, including ko01120 (Microbial metabolism in diverse environments), ko00350 (Tyrosine metabolism), ko01065 (Biosynthesis of alkaloids derived from histidine and purine), ko00020 (Citrate cycle (TCA cycle)), ko00010 (Glycolysis/Gluconeogenesis), and ko01063 (Biosynthesis of alkaloids derived from shikimate pathway). The results showed that several important metabolic pathways were enriched, involving energy metabolism, glucose metabolism, amino acid metabolism, as well as synthesis and degradation processes. This suggests that the metabolic activities in the samples may have undergone extensive and complex changes.

## 4. Discussion

Investigating the long-term effects of antimicrobial compounds and their metabolites on the quality and safety of chicken meat is crucial for safeguarding poultry product quality and consumer health. This study examined the effects of early ciprofloxacin (CIP) administration on Xueshan chickens, encompassing various aspects including growth performance, drug residues, and meat quality.

Our comprehensive analyses revealed that early CIP use significantly affected the meat quality of Xueshan chickens and induced complex metabolic responses. Regarding growth performance, our results indicated no significant difference in body weight between the CIP-treated group and the control group before reaching market age. This suggests that early CIP administration did not substantially impact the overall growth rate of Xueshan Chickens. However, significant changes were observed in meat quality parameters. Muscle pH (both pH_1_ and pH_24_) was significantly lower in the CIP-treated group, while drip loss and moisture content were significantly higher. pH is a key factor affecting meat quality and is directly related to several important quality parameters [31,32]. In the early post-mortem period, a decrease in pH typically causes muscle proteins to approach their isoelectric point, reducing their ability to bind water molecules and decreasing the muscle’s water-holding capacity [32,33]. This phenomenon explains the increased drip loss observed in our study. Moisture content is another crucial factor influencing meat quality. We observed a significant increase in muscle moisture content in the CIP-treated group, concurrent with increased drip loss. This seemingly paradoxical phenomenon reflects the complexity of meat quality [34]. The distribution of water in muscle tissue (e.g., intracellular water, extracellular water) has a significant impact on meat quality [35]. This apparent contradiction may be due to CIP treatment altering the water distribution state, a hypothesis that warrants further investigation.

The metabolomic analyses yielded particularly striking results [22]. Untargeted metabolomic analysis revealed that CIP treatment significantly altered the metabolic profile of market-age chicken. Multivariate statistical analyses clearly distinguished the metabolic patterns of CIP-treated and control groups. We identified 54 differential metabolites that demonstrated distinct metabolic differences between the two sample groups, reflecting CIP’s interference with chicken metabolic pathways. Among these metabolites, several key compounds were particularly noteworthy due to their significant alterations and potential implications for chicken physiology and meat quality. Linoleic acid, an essential omega-6 fatty acid, exhibited elevated levels in the ciprofloxacin-treated group. Previous research has demonstrated that an imbalanced ratio of omega-6 to omega-3 fatty acids, particularly an excess of omega-6, may promote inflammatory responses and increase free radical production in the body, leading to oxidative stress [36,37]. Additionally, ciprofloxacin-treated chickens had higher amounts of palmitic acid, a common saturated fatty acid. This elevation may change the meat’s fatty acid makeup, which would impact its flavor and nutritional value. According to a study by dos Santos et al. [38], variations in the firmness and juiciness of meat were linked to increased amounts of saturated fatty acids. Moreover, it has been demonstrated that palmitic acid encourages the body’s chronic inflammation, which is known to have a role in the emergence of a number of chronic illnesses. Depletion of pyridoxal phosphate, the active form of vitamin B6, was observed in the group that received ciprofloxacin treatment [39]. Since this vitamin is essential for the metabolism of amino acids, a decrease in it could have a major effect on chicken muscle growth and protein synthesis [40]. Interestingly, correlation analysis revealed significant associations among these differential metabolites, particularly between DL-vanilmandelic acid and several other metabolites in the control group. These correlations may indicate interconnected metabolic pathways, shared regulatory mechanisms, or systemic effects of CIP treatment. For instance, the correlation between DL-vanilmandelic acid and pyridoxal phosphate might reflect broader changes in amino acid metabolism networks. WGCNA revealed the modular structure and scale-independent features of the metabolites, providing a holistic view rather than focusing solely on individual metabolite changes. This approach is particularly applicable to complex biological systems, such as CIP’s effect on Xueshan chicken metabolism in this study. Correlation analysis between modules and traits identified the dark orange module as significantly correlated with the phenotype.

KEGG pathway analysis provided comprehensive information for understanding higher functions and biological systems [28,41]. Metabolic pathway network maps demonstrated complex interactions between differential metabolites, revealing multiple highly interconnected functional modules. Functional enrichment analysis and metabolic network analysis of differential metabolites and core module metabolites, respectively, revealed a common focus on ko01120 (Microbial metabolism in diverse environments) and ko00350 (Tyrosine metabolism). The enrichment of metabolites in microbial metabolic pathways suggests that CIP significantly impacts the chicken’s microbial community. As an antimicrobial agent, CIP is expected to alter microbial composition, which in turn affects the metabolic profiles of chicken meat. This observation is consistent with recent work by G. Plata et al. [8], who reported microbiological responses to antibiotics in poultry. This finding emphasizes the importance of considering microbial dynamics when assessing the impact of antimicrobial treatments of food. Enrichment of tyrosine metabolism suggests possible changes in amino acid metabolism. Tyrosine, an important amino acid involved in various physiological processes including protein synthesis and signal transduction, serves as a precursor to neurotransmitters such as dopamine and adrenaline, as well as thyroid hormones. The significant enrichment of this pathway suggests that CIP treatment may affect protein turnover and synthesis of tyrosine-derived metabolites [42]. Alterations in this pathway can impact the nutritional quality and biochemical properties of meat, potentially affecting its flavor, texture, and overall quality. These discoveries have important ramifications for poultry-farming methods. The influence of CIP on meat quality and overall metabolism is critical. Producers might need to weigh the pros and cons of CIP usage and investigate alternative disease-control methods to minimize its negative impact on meat quality. This may require enhanced collaboration among veterinarians, animal scientists, and food technologists to develop comprehensive strategies that harmonize health management with product-quality objectives.

The goal is to develop rapid biomarker-based assays to monitor CIP usage in poultry. Future studies will include additional antibiotics to better understand their role in poultry farming. This will help us better understand the roles and impacts of different types of antibiotics in poultry production, and provide a scientific basis for developing more optimal farming practices. Ultimately, it will improve the quality and safety of poultry products, while tackling the global challenge of antibiotic resistance.

## 5. Conclusions

In conclusion, this study investigated the effects of early ciprofloxacin (CIP) administration on Xueshan chickens, focusing on growth performance, meat quality, and metabolic profiles. Our findings revealed that while early CIP use did not significantly affect growth rate, it did induce specific changes in meat quality parameters (lower muscle pH, and higher drip loss and moisture content) and metabolic profiles (pathways related to Microbial metabolism in diverse environments and Tyrosine metabolism). These findings provide insights into the specific effects of early CIP administration on meat quality and metabolism in Xueshan chickens. While our results suggest potential mechanisms of CIP action, including alterations in microbial and amino acid metabolism, further research is needed to fully elucidate these mechanisms and their long-term implications for poultry production.

## Data Availability

The raw sequence data have been submitted to the National Genomics Data Center (NGDC), Beijing Institute of Genomics (BIG), Chinese Academy of Sciences (CAS) PRJCA020749.

## Supplementary Materials

The following supporting information can be downloaded at: https://www.mdpi.com/article/10.3390/ani14162395/s1, Table S1: Effect of CIP on body weight (g) of Xueshan Chicken during growth.; Table S2: Effect of CIP on meat quality of Xueshan Chickens.; Table S3: Determination of CIP content in the muscles of Xueshan chicken.; Table S4: Results of metabolite identification.; Figure S1: Quality control. Overlap map of total ions in POS (A) and NEG (B).
