# Supplementary material for: Effect of Early Ciprofloxacin Administration on Growth Performance, Meat Quality, Food Safety, and Metabolomic Profiles in Xueshan Chickens
Source: Animals (Basel). 2024 Aug 18;14(16):2395. doi: 10.3390/ani14162395 (PMC11350917; doi:10.3390/ani14162395)
Supplement: Supplementary file 1 [file animals-14-02395-s001.zip › animals-3120769-supplementary.pdf]

**Table S1.** Effect of CIP on body weight (g) of Xueshan Chicken during growth.

| Age (day) | C | T | *P* value |
|---|---|---|---|
| 14 | 177.8±17.27 | | - |
| 19 | 302.89±15.59 | 275.46±26.68 | 0.120 |
| 23 | 381.44±20.86 | 366.84±33.23 | 0.258 |
| 28 | 470.33±39.49 | 482.69±35.19 | 0.460 |
| 33 | 571.46±78.87 | 581.55±31.95 | 0.721 |
| 43 | 707.76±116.84 | 825.22±91.43 | 0.200 |
| 58 | 1205.60±119.12 | 1222.77±134.99 | 0.756 |
| 78 | 1683.60±254.97 | 1722.15±167.21 | 0.680 |
| 98 | 1976.11±294.25 | 2197.93±160.39 | 0.064 |

C: control group; T: CIP-treated group. Subsequent tables are also represented in this way.

**Table S2.** Effect of CIP on meat quality of Xueshan Chickens.

| Trait | C | T | *P* value |
|---|---|---|---|
| $pH_1$ | 6.30±0.30 | 6.00±0.26 | *0.02* |
| $pH_{24}$ | 6.39±0.27 | 5.82±0.25 | <0.01 |
| *L** | 40.00±3.19 | 38.69±4.75 | 0.31 |
| *a** | 13.26±3.41 | 12.34±3.70 | 0.4 |
| *b** | 8.23±1.85 | 7.54±1.89 | 0.25 |
| Drip loss（%） | 15.14±3.20 | 17.60±3.18 | 0.02 |
| Moisture（%） | 73.16±0.57 | 74.00±0.49 | <0.01 |
| Protein（%） | 23.96±1.45 | 23.29±1.82 | 0.12 |
| Intramuscular fat（%） | 2.57±0.32 | 2.62±0.28 | 0.6 |
| Collagen（%） | 1.38±0.25 | 1.37±0.1 | 0.87 |

**Table S3.** Determination of CIP content in the muscles of Xueshan chicken.

| Concentration (mg/kg） | C | T | *P* value |
|---|---|---|---|
| Ciprofloxacin | - | - | - |

**Table S4.** Results of metabolite identification.

| Type | All | known | unknown |
|---|---|---|---|
| POS | 9919 | 1223 | 8690 |
| NEG | 9161 | 1058 | 8103 |

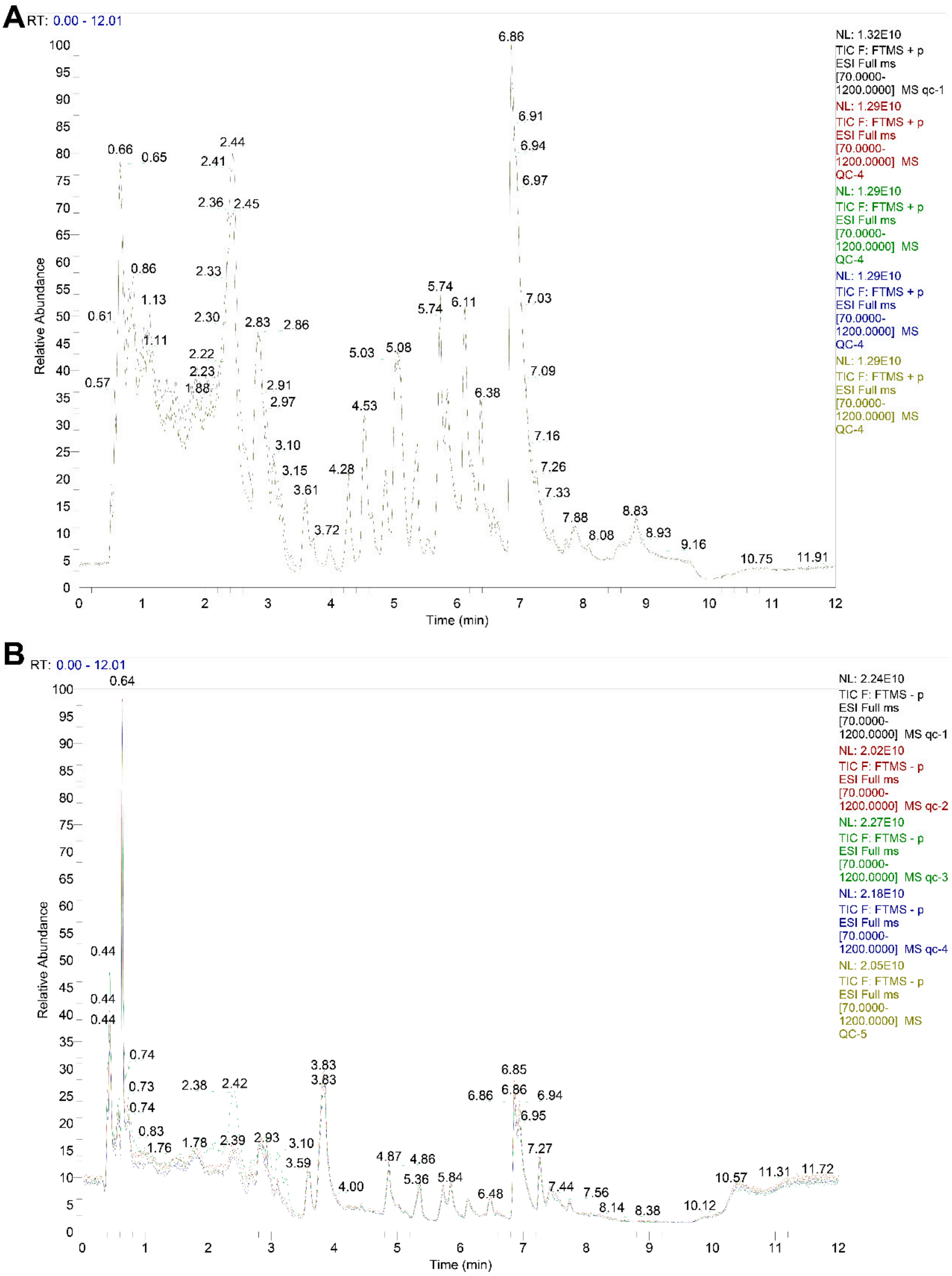


**Figure S1.** Quality control. Overlap map of total ions in POS (A) and NEG (B).
